# APASdb: a database describing alternative poly(A) sites and selection of heterogeneous cleavage sites downstream of poly(A) signals

**DOI:** 10.1093/nar/gku1076

**Published:** 2014-11-06

**Authors:** Leiming You, Jiexin Wu, Yuchao Feng, Yonggui Fu, Yanan Guo, Liyuan Long, Hui Zhang, Yijie Luan, Peng Tian, Liangfu Chen, Guangrui Huang, Shengfeng Huang, Yuxin Li, Jie Li, Chengyong Chen, Yaqing Zhang, Shangwu Chen, Anlong Xu

**Affiliations:** 1 State Key Laboratory of Biocontrol, Guangdong Province Key Laboratory of Pharmaceutical Functional Genes, School of Life Sciences, Sun Yat-Sen University, Higher Education Mega Center, Guangzhou 510006, People's Republic of China; 2 School of Basic Medical Sciences, Beijing University of Chinese Medicine, Beijing 100029, People's Republic of China

## Abstract

Increasing amounts of genes have been shown to utilize alternative polyadenylation (APA) 3′-processing sites depending on the cell and tissue type and/or physiological and pathological conditions at the time of processing, and the construction of genome-wide database regarding APA is urgently needed for better understanding poly(A) site selection and APA-directed gene expression regulation for a given biology. Here we present a web-accessible database, named APASdb (http://mosas.sysu.edu.cn/utr), which can visualize the precise map and usage quantification of different APA isoforms for all genes. The datasets are deeply profiled by the sequencing alternative polyadenylation sites (SAPAS) method capable of high-throughput sequencing 3′-ends of polyadenylated transcripts. Thus, APASdb details all the heterogeneous cleavage sites downstream of poly(A) signals, and maintains near complete coverage for APA sites, much better than the previous databases using conventional methods. Furthermore, APASdb provides the quantification of a given APA variant among transcripts with different APA sites by computing their corresponding normalized-reads, making our database more useful. In addition, APASdb supports URL-based retrieval, browsing and display of exon-intron structure, poly(A) signals, poly(A) sites location and usage reads, and 3′-untranslated regions (3′-UTRs). Currently, APASdb involves APA in various biological processes and diseases in human, mouse and zebrafish.

## INTRODUCTION

Polyadenylation processing of pre-mRNAs is an essential step in the generation of mature mRNAs, and involves the coupling of site-specific cleavage and addition of a polyadenylated tail at the 3′-end of nascent mRNAs (1,2). Polyadenylation not only impacts the stability and export of mature mRNAs from the nucleus (3–5), but also contributes to the translation initiation and efficiency in the cytoplasm (6,7). The use of alternative polyadenylation (APA) cleavage sites for different biological processes and diseases allows a single gene to encode multiple mRNA transcripts variable in length, particularly in the 3′-untranslated regions (3′-UTRs). The cellular APA mechanism may change the final protein sequence due to cleavage sites located in the intron or internal exons. Tandem APA-sites in the last exon of mRNAs can be alternatively used in polyadenylation in much higher frequency to generate different tandem 3′-UTRs in length (8–10). Recently, tandem 3′-UTRs have been revealed to play increasingly important roles in regulating gene expression networks because they enable the loss and gain of *cis*-regulatory elements in 3′-UTRs of nascent mRNAs in different biological conditions and diseases, notably the microRNA seed sites and other binding sites of transcriptional factors (9,11–13). Thus, a database describing the precise map and usage quantification of different APA sites on a genome-wide scale for all genes is urgently needed for better understanding the APA-directed regulation of gene expression for a given biological process.

Several APA-related existing databases, such as polyA_DB2 and polyCdb, use transcript–genome alignments and expressed sequence tags (ESTs) to identify and characterize putative 3′-processing sites (14–16). Thus, they are more restrictive in data scale and 3′-processing site identification due to the limited accumulation of cDNAs and ESTs, especially the lack of poly(A) sites located in introns and internal exons that may change the protein sequences. Another, UTRdb, is based on the careful parsing of EMBL/GenBank records, and focuses on sequences but not on 3′-processing sites and the corresponding poly(A) signals on a genome-wide scale (17). The previous polyA-seq data available as UCSC tracks, including the recent APADB, utilize the next-generation sequencing (NGS) technology to identify 3′-ends, but they contains limited APA-datasets derived from human tissues and lack for datasets on human diseases, especially the datasets on embryogenesis of zebrafish model organism (18,19).

Here we present a new web-accessible database of APA sites on a genome-wide scale, named APASdb, based on the APA datasets deeply profiled by sequencing alternative polyadenylation sites (SAPAS) method reported previously (8,9,20). The NGS-coupled SAPAS method is as accurate as the existing RNA sequencing (RNA-seq) approaches for digital gene expression. SAPAS is capable of high-throughput sequencing and quantifying the 3′-ends of polyadenylated transcripts, and further identifying the location and usage of different APA sites located in introns and internal exons as well as another kind of tandem APA sites in the last exon. Therefore, APASdb has near perfect coverage for poly(A) sites of a whole-genome, and details all the heterogeneous cleavage sites downstream of each poly(A) signal (21,22), making it much better than the previous databases generated only using alignments of limited cDNAs and ESTs to a genome to identify the putative 3′-processing sites. Moreover, APASdb enables the usage quantification of different poly(A) sites on a genome-wide scale by computing their corresponding reads after normalization. As a web-accessible database, APASdb provides convenient URL-based retrieval, browsing and presentation of several types of information on line, including exon-intron structure, poly(A) signal types and positions, poly(A) sites locations and usage reads, and 3′-UTR regions. Also, the APA data can be displayed in their genomic context via a popular genome browser (Gbrowse) (23–25). Currently, APASdb contains kinds of APA datasets from three model organisms, human (*H. sapiens*), mouse (*M. musculus*) and zebrafish (*D. rerio*). They detail APA sites in different types of cells, tissues, organs and embryos, as well as tissues in a variety of physiological and pathological conditions. However, as more datasets are generated with the SAPAS method, APASdb is expected to be increasingly valuable for researchers to study polyadenylation mechanisms and APA-mediated gene regulation and other biological functions.

## MATERIALS AND METHODS

### SAPAS library preparation

**Figure 1.**
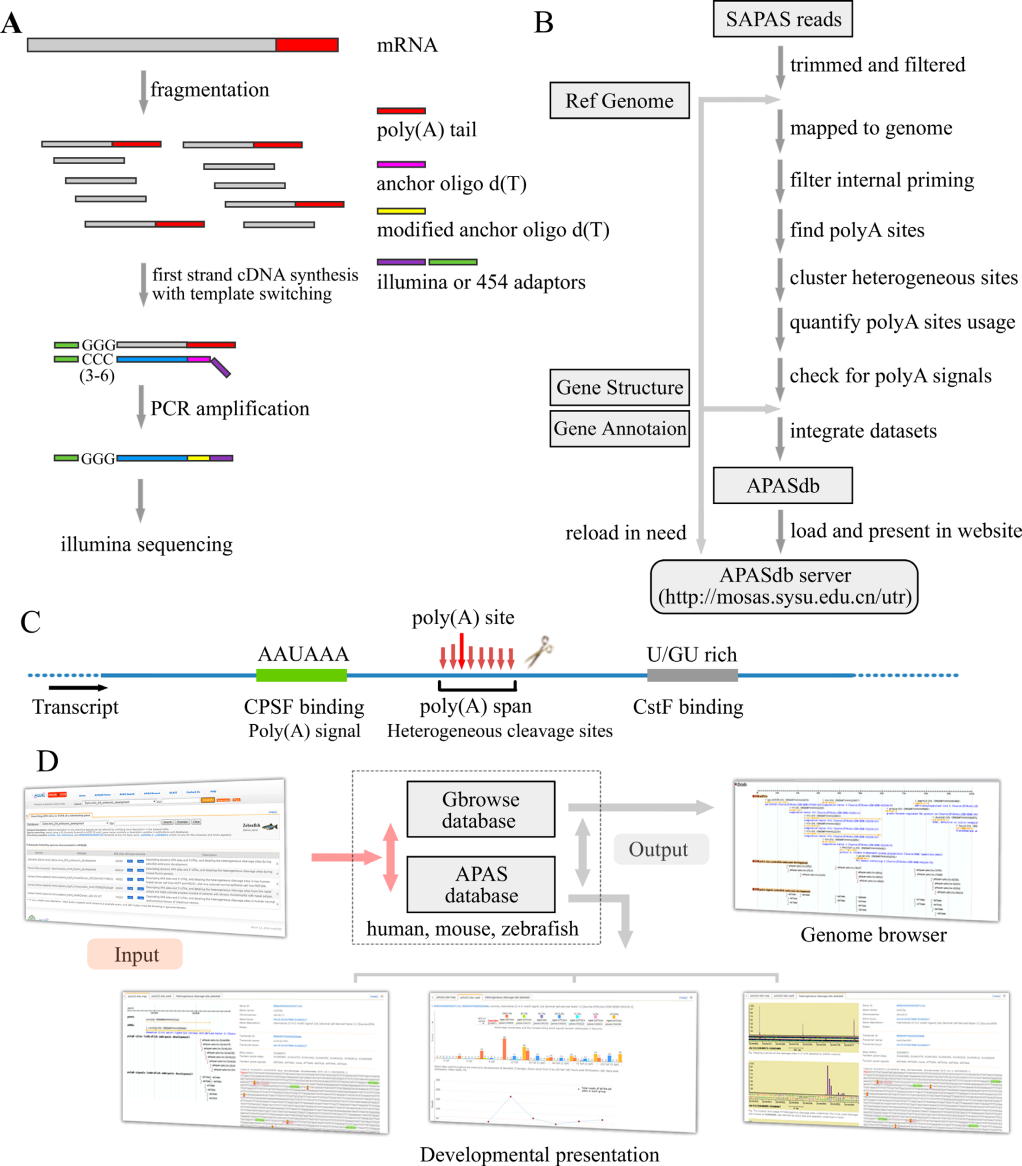
Overview of the APASdb website. (**A**) Experiment outline of SAPAS library preparation. (**B**) Outline of the APASdb building pipeline. The data flow is indicated by arrowed lines. Data generated by this optimized pipeline, contains positions and reads of heterogeneous cleavage sites, poly(A) signals and 3′-UTR sequences, as well as the locations and usage reads of poly(A) sites. (**C**) Schematic representation of a poly(A) site and polyadenylation configuration. A poly(A) span is a cluster containing heterogeneous cleavage sites (arrowed lines) and the most-frequently used cleavage site is defined as the reference point for a poly(A) site. The binding sites for the cleavage polyadenylation specificity factor (CPSF) and cleavage stimulatory factor (CstF) are also depicted.(**D**) Architecture of the APASdb website. Arrows denote the direction of information flow, and several output pages are shown, including the popular genome browser (Gbrowse), especially the developmental presentation termed ‘poly(A)-site map’, ‘poly(A)-site usage’ and ‘heterogeneous cleavage-site selection’.

Several sequencing libraries (Supplementary Notes), were prepared as described previously (8). Briefly, as indicated (Figure 1A), total RNA was respectively extracted from different samples by TRIzol reagent (Invitrogen), and about 10 μg of total RNA was randomly fragmented by heating. An anchored oligo d(T) primer and a 5′-template switching linker tagged with Illumina adaptors were used in template switch reverse transcription by SuperScript II reverse transcriptase (Invitrogen). Two mutations in the poly(A) were introduced by polymerase chain reaction (PCR) amplification with a determined number of cycles to ensure that the double strand cDNA remain in the exponential phase of amplification. The PCR products with a size of 250–400 bp were recovered by polyacrylamide gelelectrophoresis gel-excision and quantified by a Qubit 2.0 Fluoromete. The average size was determined by Agilent 2100 bioanalyzer. As followed, a quality control was carried out by plasmid recombinant and Sanger sequencing. The recovery was ligated to pGEM-T vector and transformed into *Escherichia coli* DH5a competent cells. Plasmid DNA was extracted and sequenced by ABI 3730 DNA Analyzer. Each end of the insert should be illumina sequence primer. The insert with long poly(A) stretch should be <5%, and most of the inserts should be mapped to the corresponding genome.

### Pipeline and raw sequences

We designed a computational pipeline (Figure 1B, further details in Supplementary Methods), to accurately map and quantify usage of different poly(A) sites on a genome scale, profiled by the SAPAS method. In summary, we first filtered Illumina-sequenced SAPAS reads to discard the reads with unrecognizable linker sequence, and trimmed to remove the linker and the ‘T's that just followed the linker until a not-‘T’ was met. If the length of a trimmed read was <25 nt, we discarded the read too. We then aligned all qualified reads to the corresponding genome using *Bowtie* software, version 0.12.5 (26). For internal priming filtering, we used the uniquely mapped reads by detecting the downstream genomic sequence 1 to 20 of cleavage sites as previously (8), that is, the read was regarded as an internal priming candidate if this 20-nt region contained more than 12 ‘A's or one of the following patterns: 5′-AAAAAAAA-3′ and 5′-GAAAA+GAAA+G-3′, where ‘+’ means ‘or more’. We defined cleavage sites by iteratively clustering the reads, locating tailing ends within 24 nt from each other and which were also aligned to the same strand of a chromosome. Cleavage clusters with two or more normalized reads were taken as poly(A) sites, and we searched for the corresponding poly(A) signals within the upstream sequence 1 to 50 nt from each poly(A) site. Using the gene structure and annotation from bioinformatics sites such as Ensembl and UCSC (27,28), we annotated the poly(A) sites and corresponding poly(A) signals.

Based on this pipeline, we processed SAPAS raw reads of samples from three model organisms, zebrafish (*D. rerio*), mouse (*M. musculus*) and human (*H. sapiens*), to generate poly(A) site datasets. These raw reads of samples (see details in Supplementary Notes), invole in zebrafish embryos in various development stages from 0 h post fertilization (hpf) to 5 day post fertilization (dpf), mouse thymic development from 15.5 days post fertilization (dpf) to 90 days post parturition (dpp), human normal 22 tissues (brain, lung, thyroid, spleen, stomach, kidney, cervix, heart, lymph node, placenta, uterus, bladder, breast, prostate, liver, pancreas, small intestine, thymus, adipose, skeletal Muscle, ovary and testicle), human breast cancer and normal cells, human carcinomatous and normal tissues of intestinum rectum, as well as the nasal polyps and nasal uncinate process mucosa of chronic rhinosinusitis patients with nasal polyps.

### Database and website design

Based on the APA-site datasets deeply profiled by the SAPAS strategy, the APASdb website is developed with open source technologies. The datasets of samples involved in related cell and tissue types or specific physiological and pathological conditions, were adopted and integrated into a larger searching dataset (Table 1), to facilitate the query and comparison display of poly(A) sites of genes in our website. For example, the searching dataset named Zv9_embryonic_development, integrating eight subsets across all the major developmental stages, describes the dynamic usage of APA-sites in zebrafish embryogenesis. Various types of information regarding locations and normalized usage reads of poly(A) sites, poly(A) signal types and positions, 3′-UTR regions and exon-intron structure of genes in the APASdb, are stored in a relational database using MySQL. Web-based HTML interactive interfaces combined with JAVA, PERl and PHP scripts provide access to the database. GD modules of PHP and Bioperl modules are used for dynamic and graphical representation (29).

**Table 1.** The searching datasets listed by species in APASdb website

Species	Searching datasets	subsets^a^	Poly(A) sites	Simple descriptions^b^
*D. rerio*	Zv9_embryonic_development	8	108 290	Dynamic APA sites and 3′-UTRs, selection of heterogeneous cleavage sites during zebrafish embryonic development.
*M. musculus*	mm9_thymic_development	8	226 858	Dynamic APA sites and 3′-UTRs, selection of heterogeneous cleavage sites in mouse thymopoiesis.
*H. sapiens*	hg19_breastCancer_MCF10A-MCF7-MB231	3	46 531	Genome-wide APA sites and 3′-UTRs, selection of heterogeneous cleavage sites in human breast cancer cell lines MCF7 and MB231, also one cultured normal epithelial cell line MCF10A.
*H. sapiens*	hg19_rectalCancer_12N-VS-12T	2	74 116	Genome-wide APA sites and 3′-UTRs, selection of heterogeneous cleavage sites in human normal and tumorous tissues of intestinum rectum.
*H. sapiens*	hg19_rhinosinusitis_11N11P25N25P26N26P	6	83 641	Genome-wide APA sites and 3′-UTRs, selection of heterogeneous cleavage sites in nasal polyps and nasal uncinate process mucosa of eosinophilic chronic rhinosinusitis patients with nasal polyps.
*H. sapiens*	hg19_human-all22-tissues	22	179 532	Genome-wide APA sites and 3′-UTRs, selection of heterogeneous cleavage sites in human 20 tissues.

^a^Total number of subsets integrated into a searching dataset.

^b^Detail descriptions of experimental samples can be referred (Supplementary Notes, or http://mosas.sysu.edu.cn/utr/search_APASdb.php?show=1).

## RESULTS

### Datasets in APASdb

As listed (Table 1), currently APASdb contains APA-site datasets involved in various cell and tissue types, or physiological and pathological conditions in three important models, human, mouse and zebrafish. The statistics and analysis of APA sites in these searching datasets seem to indicate that, APASdb not only keeps near perfect coverage of poly(A) sites but also contains much more novel poly(A) sites in the above-mentioned species (Supplementary Table S1, Supplementary Figure S1-1 to S1-6). Here, we only take the searching dataset (named Zv9_embryonic_development) for detail discussion. This dataset consists of eight sample-subsets across all the major developmental stages in zebrafish embryogenesis, including 59 294 885 reads (obtained after mapping and filtering), and nearly 90% of them were mapped to annotated 3′-UTRs or 1-kb downstream regions (Supplementary Figure S1-1, left). Of the 108 290 poly(A) sites across all stages with five or more normalized reads, 12% were mapped to the known Ensembl transcription termination sites (TTS), and the remaining 88% were unreported previously, especially 23.5% and 14.6% were mapped to the 3′-UTR and 1-kb downstream from the Ensembl canonical genes, respectively (Supplementary Figure S1-1, right). The authenticity of the novel poly(A) sites was also checked by 3′RACE in our previous report (9), of 30 novel poly(A) sites (five sites for each of the location categories shown in Supplementary Figure S1–1, excluding Ensembl TTS), 28 (93%) were validated. Also, the previous comparison of our data with another RNA-seq dataset (9,30), showed that most (>70%) intergenic poly(A) sites were located within 5-kb downstream from RNA-seq reads, indicating the authenticity of these novel poly(A) sites. APASdb is intended to broaden the known poly(A) site coverage with growing numbers of APA-site datasets generated with the SAPAS method. These APA data are available in our APASdb website, and summarized in graphical representations for quantification and comparison of APA sites used in different biological processes and/or diseases.

### General organization and access of APASdb

The general organization of the APASdb website is presented (Figure 1D), and the APA datasets are available from our APASdb web server addressed at http://mosas.sysu.edu.cn/utr. Data can be quickly queried and presented by user's keywords in the web-query interface, where gene annotations were loaded as well as the gene-related poly(A) sites and poly(A) signals are highlighted in the corresponding genome sequence, including the heterogeneous cleavage sites clusters (indicated in Figure 1C). The interface dynamically creates a graphic to track them together with the corresponding exon-intron structure of transcript variants of the searched gene, not only detailing the location and quantification of the heterogeneous cleavage sites downstream of each poly(A) signal, but also adding the usage quantification of APA sites and the expression pattern of corresponding gene in various cells, tissues and organs, or in different biological situations and diseases. The APA datasets were integrated to Gbrowse database, so as to provide an interactive and graphical view of APA sites associated with genomes, genes, transcripts and transcript annotations on a genome-wide scale.

### Searching APASdb

**Figure 2.**
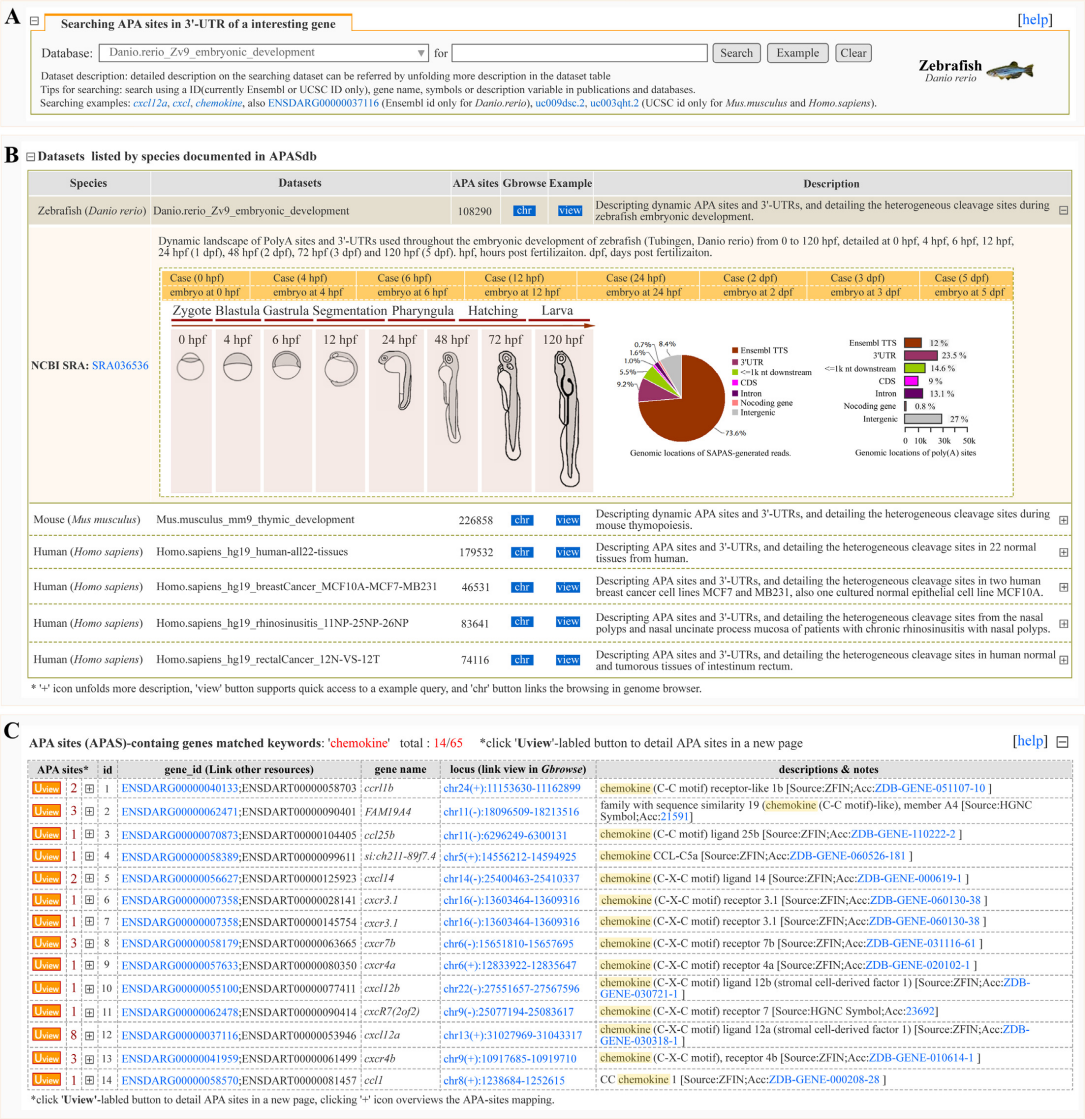
Screen shot of the searching page and the media page resulting from a fuzzy query keyword of ‘chemokine’. (**A**) User retrieval interface designed to query datasets. (**B**) Descript list of datasets in retrieval interface. The List summarizes the released datasets and directs user's query. The ‘view’ button supports quick access to a example query of dataset and the ‘chr’ button links the browsing of dataset in a genome browser (Gbrowse). (**C**) List of APA sites-contained genes matching the fuzzy keyword of ‘chemokine’. Each icon displayed in ‘APAS’ column of the result table links a detail page to show more corresponding information of APA sites and the number highlighted in ‘APAS’ column indicates the number of APA-sites located in the transcript locus, and texts with hyperlinks in other columns enable redirecting to other extensive resources, especially the texts in ‘locus’ column guide user to the specified URLs to browse APA sites associated with genes in a genome browser. For direct viewing the example mentioned here, the reader is asked to refer to http://mosas.sysu.edu.cn/utr/search_APASdb.php?seqkeywords=chemokine.

Our ‘APAS Search’ feature is designed to search the interesting datasets in APASdb and present APA information of a user's genes of interest according to the searching tips. Currently, Ensembl id (for *D. rerio*) and UCSC id (for *M. musculus* and *H. sapiens*) are allowed for precise query, also fuzzy query by using keywords such as gene name, symbol and simple description is permitted. Clicking the button labeled ‘Example’ yields an example keyword suited for searching a selected dataset (Figure 2A), and the subsets and detail descriptions on the searched dataset can be unfolded by clicking the corresponding ‘+’-labeled icon (Figure 2B). Fuzzy search, using a fuzzy keyword of ‘chemokine’, may lead to a media page to list all the matched APA sites-contained genes in a dynamic table (Figure 2C), so as to facilitate the selective view of their corresponding APA information in a linked detail page (described next), but searching by using a precise keyword can give users quick access to the detailed page to view various types of information on APA sites and the related graphics.

### Graphical display of APA sites of a queried gene

Under the ‘poly(A)-site map’ tab in the detailed page (Supplementary Figure S2), there is a summary for the queried gene, including the corresponding APA sites and poly(A) signals mapped to the searched transcript locus (containing 5′ and 3′ flanking region of 1 kb). Particularly, the APA sites, UTR region and exon-intron structure of transcript variants of the queried gene are graphically presented in a proper scale, which enables tracking them together with the corresponding genome. Here, we take the chemokine (c-x-c motif) ligand 12a (*cxcl12a*) for example. The unfolded panel labeled ‘pooled’ shows all the APA-sites of *cxcl12a* appeared in zebrafish embryogenesis. Total eight APA sites are detected and seven poly(A) sites (pA:31040950, pA:31041075, pA:31041303, pA:31042052, pA:31042222, pA:31042612 and pA:31043309) are in the annotated 3′-UTR, including one poly(A) site (pA:31043625) located in the 1-kb downstream regions. The poly(A) site (pA:31041303) in 3′-UTR has no poly(A) signal, but each of the rest poly(A) sites has at least a corresponding poly(A) signal. Clicking ‘+’-icons on the folded panels (labeled 0 hpf, 4 hpf, 6 hpf, 12 hpf, 24 hpf, 48 hpf, 72 hpf and 120 hpf respectively), selectively observes and compares the APA-sites of *cxcl12a* appeared in the different stages of zebrafish embryogenesis (Supplementary Figure S2, left). In order to facilitate manual checking of the cleavage sites in the corresponding genome sequence, all the detected heterogeneous cleavage sites clustered to a poly(A) site are underlined and highlighted in red, and their upstream poly(A) signals if exists are highlighted in green. Also, the searched transcript locus is indicated, including the marked exons (light gray background with a brown font), introns and UTRs (light gray background with a green font). Especially, the most-frequently used cleavage site defined as the reference poly(A) site in each cluster, is specially highlighted in dark red and underlined in bold (Supplementary Figure S2, right).

### Quantification of APA sites and expression pattern of a gene

**Figure 3.**
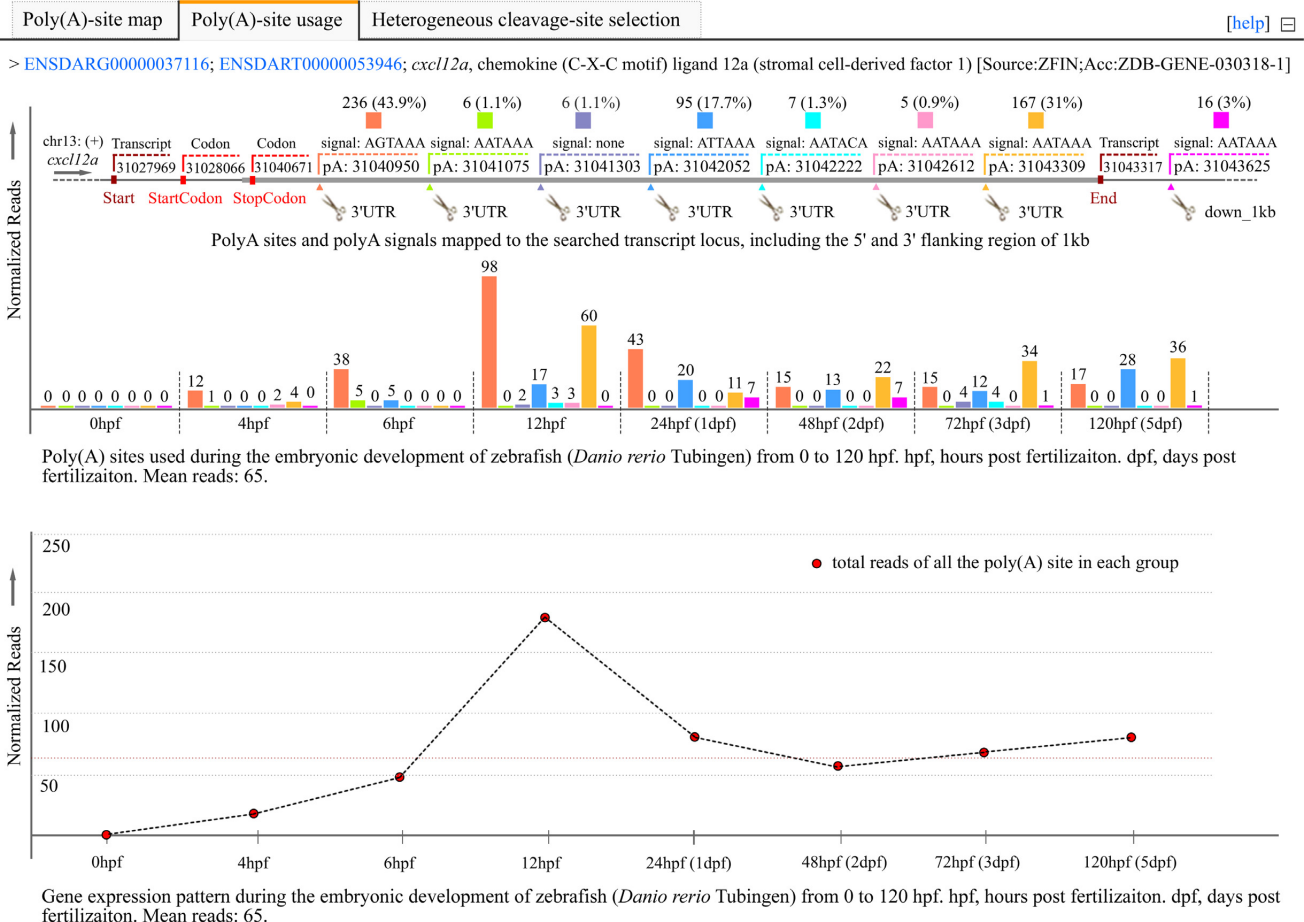
Screen shot of the detail page with the unfolded ‘polyA-site used’ tab to reveal the dynamic usage of APA sites and expression pattern of *cxcl12a* in zebrafish embryogenesis. The bar chart indicates the location and usage quantification of APA sites of *cxcl12a* from 0 hpf to 5 dpf, and by summing the normalized supporting reads of APA sites appeared in each stages, the curve diagram presents the expression pattern of *cxcl12a* in zebrafish embryogenesis. *Y-axis*, numbers of normalized reads. *Reads*, read number normalized to per million mapped read; *hpf*, hours post fertilization;* dpf*, days post fertilization;*pA*, poly(A) sites. For browsing the example described here, readers are asked to refer to http://mosas.sysu.edu.cn/utr/search_APASdb.php?seqkeywords=ENSDARG00000037116.

Clicking the ‘poly(A)-site used’ tab in the detailed page (Figure 3), draws another two matched graphics dynamically. One is a bar chart indicating the usage quantification of APA sites of queried gene in the related-subsets integrated into the searched dataset and the other is a curve diagram created to show the expression pattern of queried gene represented by the sum of supporting reads of corresponding APA sites. For the example query of *cxcl12a*, the bar chart shows usage quantification of eight poly(A) sites in the embryonic development of zebrafish (0 hpf, 4 hpf, 6 hpf, 12 hpf, 24 hpf, 48 hpf, 72 hpf, 120 hpf), including seven poly(A) sites (pA:31040950, pA:31041075, pA:31041303, pA:31042052, pA:31042222, pA:31042612 and pA:31043309) in the annotated 3′-UTR and the last poly(A) site (pA:31043625) located in downstream region of 1 kb (down_1 kb). Obviously, in earlier embryos within 12 hpf, the poly(A) site of pA:31040950 is predominantly used and keeps *cxcl12a* transcripts with the shortest 3′-UTRs. After 12 hpf, multiple poly(A) sites are adopted to generate transcript variants with longer 3′-UTRs, especially the frequently used poly(A) site of pA:31042052 and pA:31043309. The transcripts with the longest 3′-UTR first appear between 24 to 48 hpf, resulting from the usage of pA:31043625 in downstream region of 1 kb (Figure 3, up). In addition, summing the normalized reads of all the 3′-ends of polyadenylated transcripts appeared draws the curve diagram to show the expression of *cxcl12a* across all the major stages of zebrafish embryogenesis. It demonstrates that the expression of *cxcl12a* increases first and gets to the maximum value at 12 hpf, then decreases quickly. After 48 hpf, the *cxcl12a* expression keeps increasing slowly (Figure 3, down).

### Detailing the selection of heterogeneous cleavage sites downstream of poly(A) signals

Clicking the ‘heterogeneous cleavage-site selection’ tab in the detailed page (Supplementary Figure S3), creates a series of figures to detail the location and usage quantification of the heterogeneous cleavage sites downstream of each poly(A) signal in a queried gene. Here, we take the example query of *cxcl12a* for detailed description. The first figure is an overview of all the cleavage sites mapped to *cxcl12a* locus and eight different read-clusters of the heterogeneous cleavage sites are indicated in dashed frames. These read-clusters direct correspond to the poly(A) sites (pA:31040950, pA:31041075, pA:31041303, pA:31042052, pA:31042222, pA:31042612 and pA:31043309) in the annotated 3′-UTR, including the last poly(A) site (pA:31043625) located in downstream 1-kb region (Supplementary Figure S3A). Also, clicking on this figure, loads a new page to observe the dynamic change of these detected read-clusters in the different stages of zebrafish embryogenesis (Supplementary Figure S4). Following the above-mentioned figure, additional eight figures are drawn to zoom in these corresponding clusters. These figures can further detail the heterogeneous cleavage sites clustered to each poly(A) site, including the usage frequency of each cleavage site in a cluster (Supplementary Figure S3B, I to VIII). To facilitate manual checking, the heterogeneous cleavage sites downstream of a poly(A) signal are underlined in a cluster. Also, the most frequently used cleavage site that has the maximum reads in a cluster, is defined as a poly(A) site and specially underlined in bold. The sum of normalized reads for all the heterogeneous cleavage sites in a cluster indicates the usage of this poly(A) site. Especially, clicking on these figures can load the new pages to detail the selection of heterogeneous cleavage sites downstream of their corresponding poly(A) signal in zebrafish embryogenesis (Supplementary Figure S5 to S12).

### Dynamic and graphical browsing of APA sites

Based on the integration of APASdb and Gbrowse database, via a genome browser (Gbrowse), APASdb websites provides dynamic browsing of APA sites associated with genomes, genes, transcripts and annotations on a genome-wide scale (‘APAS Browse’ feature). The selective view in three layers, such as overview, region and details, are provided for browsing APA sites in several reference genomes, including human (GRch37/hg19), mouse (NCBI37/mm9) and zebrafish (Zv9/danRer7). One or more APA datasets from these species can be quickly loaded and graphically browsed online, by clicking the ‘Update image’ button after selecting the corresponding checkboxes of datasets in ‘Tracks’ panel (Supplementary Figure S13, bottom). This not only enables an overall picture of APA sites in certain cells, tissues and organs, or in a variety of physiological and pathological conditions, but also offers a more direct way to compare the usage of APA sites and further find the general and specific poly(A) sites. Here, we give an example for APA sites of G protein-coupled receptor 126 (*GPR126*) in human breast cancer cell lines MCF7 and MB231, normal breast tissue and rectal cancer tissue. As tracked and indicated in detailed layers, the poly(A) sites (pA:142767384, pA:142767390 and pA:142767391) are located within 24 nt from each other in the same strand, so they are usually taken for a same poly(A) site, seeming to be the general poly(A) sites for human breast cancer and rectum cancer. The poly(A) sites (pA:142765090 and pA:142767294) may be specific in human breast tissue, especially, the poly(A) site (pA:142767294) appeared only in human breast cancer cell lines MCF7 and MB231 (Supplementary Figure S13, middle).

## DISCUSSION

We present a comprehensive database of APA sites in human, mouse and zebrafish based upon a developed NGS-dependent 3′-end sequencing strategy, namely, SAPAS. Thus, in a sense, we provide additional experimental support for poly(A) sites in the polyA_DB2 and transcript termination database of UCSC and Ensembl. It seems that APASdb not only contains much more novel poly(A) sites, but also has near perfect coverage for APA sites of genes throughout human, mouse and zebrafish (Supplementary Table S1, Supplementary Figure S1-1 to S1-6). At present, our APASdb are focused on the dataset generated with the SAPAS strategy, broadening the poly(A) site coverage with growing numbers of APA datasets. Also, the publicly available poly(A) data generated by the other NGS-based protocols, such as polyA-seq (19) and massive analysis of cDNA ends (MACE) (18,31), will be selected and added into our website to extend the usefulness of APASdb in the future. Actually, APASdb has more comprehensive APA datasets for human. It not only has subsets (total 11) involved in human diseases, but also keeps full subsets involved in human 22 normal tissues, much more than the polyA-seq data (only five tissues) and MACE data (only seven tissues). These subsets simultaneously detail the location and usage of APA sites and facilitate the analysis of tissue-specific APA sites in human tissues. Especially, the disease-related subsets display and compare the APA sites between human normal and tumor cells or tissues, such as breast cancer, rectal cancer and eosinophilic chronic rhinosinusitis with nasal polyps, so as to promote the investigation of diseases-related APA site switching. Also, there are eight subsets integrated to indicate the changes of APA sites in mouse thymic development, helping identifying the APA-site switching involved in vertebrate thymopoiesis. For the zebrafish model often used in genetics, eight subsets across all the major stages of embryogenesis are adopted and combined to profile the dynamic usage of APA sites and contribute to the understanding of tandem 3′-UTR regulation in the control of vertebrate embryogenesis.

We also frequently observed that multiple cleavage sites downstream of a poly(A) signal were only a few nucleotides apart, an interesting phenomenon usually called heterogeneity (21,22,32). APASdb details the heterogeneous cleavage sites for all genes in a genome-wide fashion and compares the variation of heterogeneous cleavage sites clustered to a poly(A) site in various cells and tissues, or in a variety of physiological and pathological conditions. This may help studying the mechanism of polyadenylation, in particular, the selection of heterogeneous cleavage sites at a given time for a given 3′-end formation.

Overall, APASdb makes it possible to identify the condition-specific poly(A) sites, helpful to studying APA-site switching mechanism and function, especailly to looking for the loss and gain of miRNA binding-sites in the dynamic 3′-UTRs. Also, APASdb simultaneously presents the expression and position of APA sites and enables the identification of APA-site switching in association with many biological processes and diseases. As a user-friendly web database, APASdb will be an increasing valuable resource for the polyadenylation and 3′-UTR research community, especially for the studies on polyadenylation mechanisms and APA-mediated gene regulation, requiring identification of poly(A) sites and indication of their corresponding conditional usage in a large dataset.

## SUPPLEMENTARY DATA

Supplementary Data is available at NAR Online.
